# Evaluation of microcirculation before and during continuous renal replacement therapy and the impact of dose prescription

**DOI:** 10.1186/cc10979

**Published:** 2012-03-20

**Authors:** C Pipili, CS Vrettou, S Poulaki, A Papastylianou, M Parisi, ES Tripodaki, S Ioannidou, S Kokkoris, E Douka, S Nanas

**Affiliations:** 1 Aretaieion University Hospital, Athens, Greece; 2 National and Kapodistrian University of Athens, Greece; 3 Evaggelismos Hospital, Athens, Greece

## Introduction

Microcirculation (MC) might provide evidence for the solute exchange taking place during the dialysis process. Near-infrared spectroscopy (NIRS) with combination of vascular occlusion technique (VOT) allows evaluation of peripheral tissue oxygen utilization and restoration mainly depending on integrity and functionality of vascular endothelium. Our purpose was to evaluate the acute effect of continuous renal replacement therapy (CRRT) on the MC as assessed by NIRS and VOT and to explore the impact of delivered CRRT dose on MC alterations.

## Methods

A total of 43 critically patients who underwent CRRT were eligible to participate in the study. The mean age of our population was 66 ± 17 years and 40% were females. The APACHE II score was 20 ± 6, the mean serum creatinine before the CRRT initiation was 2.6 ± 1.6 mg/dl and the mean CRRT delivered dose was 23 ± 6 ml/kg/hour. The median value of dose was used to form groups of high (>22.5 ml/kg/hour) and low (≤22.5 ml/kg/hour) delivered dose. NIRS parameters were evaluated before CRRT initiation (H0), at 6 hours (H6) and at 24 hours (H24) during the CRRT process. Tissue oxygen saturation (StO_2_, %), defined as the percentage of hemoglobin saturation in the microvasculature compartments, was measured with a probe placed on the thenar muscle. A 3-minute brachial VOT was applied to evaluate the oxygen consumption rate (OCR, %/minute), the recovery slope (RS, %/minute), and the hyperemia recovery area as the area (units/minute) under the StO_2_% curve above baseline values.

## Results

Two-way repeated-measures ANOVA were performed for StO_2_, OCR, RS and hyperemia recovery area at H0, H6 and H24. StO_2 _correlated with RIFLE on admission and at the time of CRRT initiation (*r *= 0.283, *P *= 0.03 and *r *= 0.45, *P *< 0.0001 respectively). There was a significant decrease in OCR with time (hours on CRRT process) (within-subjects ANOVA *F *= 4.83, *P *= 0.014) and especially between H0 and H24 (-10.5 ± 9.4 vs. -12 ± 8.3, *P *= 0.008). Furthermore, a significant increase in RS was found in patients who received a high CRRT dose (between-subjects ANOVA *F *= 4.5, *P *< 0.05), especially at H6 post CRRT initiation (76 ± 117 vs. 86 ± 128, *P *= 0.05).

## Conclusion

Critically ill patients, receiving a dialysis dose higher than 22.5 ml/kg/hour, showed improved MC. Further studies are needed to investigate the role of NIRS technology as a tool to assess the need for CRRT initiation in acute renal failure.

